# Policy implementation for methicillin-resistant *Staphylococcus aureus* in seven European countries: a comparative analysis from 1999 to 2015

**DOI:** 10.1080/20016689.2017.1351293

**Published:** 2017-07-26

**Authors:** Takuya Kinoshita, Hironobu Tokumasu, Shiro Tanaka, Axel Kramer, Koji Kawakami

**Affiliations:** ^a^ Department of Pharmacoepidemiology, Graduate School of Medicine and Public Health, Kyoto University, Kyoto, Japan; ^b^ Institute of Hygiene and Environmental Medicine, University Medicine Greifswald, Greifswald, Germany

**Keywords:** Infection control, antimicrobial drug resistance, surveillance, healthcare-associated infections, MRSA, health policy, mandatory surveillance, LA-MRSA

## Abstract

**Background**: Policies to reduce methicillin-resistant *Staphylococcus aureus* (MRSA) infections, both healthcare-acquired (HA-MRSA) and livestock-associated (LA-MRSA) are implemented Europe-wide, but evaluations are difficult for countries yet to implement such policies. A descriptive study was conducted, describing multinational MRSA rates and policy implementation, focusing on MRSA mandatory surveillance. We also investigated antibiotic use and MRSA rates and the use of veterinary antibiotics.

**Methods**: This study used Europe-wide surveillance data on infectious diseases (EARS-Net), antibiotic consumption (ESAC-Net), and veterinary medicine (ESVAC). We visualized LA- and HA-MRSA related policies and MRSA rates from 1999 to 2015 in seven European countries. Changes in MRSA rates after implementation of an MRSA mandatory surveillance policy were investigated by setting each country as rate of 1.0 and compared countries with and without such policy. Correlations between antibiotic use and MRSA rates from 1999 to 2012 were investigated using defined daily dose. Sales data were used to investigate veterinary antibiotic use.

**Results**: MRSA rates were 1–45.4% across the seven countries between 1999 and 2015. MRSA rates changed between 0.61 and 0.24 after the implementation of mandatory surveillance policies within a 6–12 year span. The rate of decrease rate in implemented and non-implemented countries ranged from 10% in Spain to 76% in the UK. The correlation between MRSA rate and cephalosporin consumption was *r *= 0.419, and for fluoroquinolones *r *= 0.305. Mean annual sales of veterinary cephalosporin and quinolone antibiotics were lowest in the UK (0.8 mg/PCU) and highest in Spain (9.7 mg/PCU) between 2009 and 2014.

**Conclusions**: There were similar but different health policy implications in the seven countries regarding LA- and HA-MRSA. Although causation could not be defined, some policies such as mandatory surveillance may be helpful for countries that have yet to implement an MRSA policy. Further investigations are needed to evaluate each policies.

## Introduction

Many countries have implemented a range of policies to reduce the incidence of methicillin-resistant *Staphylococcus aureus* (MRSA) infections and thereby safeguard limited healthcare resources. MRSA was discovered in 1961 after the introduction and widespread use of penicillin to treat bacterial infections [1]. MRSA infections not only increase the possibility of medical complications but also place a strain on hospital resources and increase medical expenses [2]. Studies have shown that the overuse of antibiotics, such as cephalosporin and fluoroquinolone, correlates with an increased risk of MRSA infections [3,4].

Therefore, three Europe-wide surveillances were established to collect antimicrobial resistance-related data: (1) the European Antimicrobial Resistance Surveillance Network (EARS-Net), collecting healthcare-acquired MRSA (HA-MRSA) incidence data; (2) the European Surveillance of Antimicrobial Consumption Network (ESAC-Net), collecting antibiotic consumption data in healthcare; and (3) the European Surveillance of Veterinary Antimicrobial Consumption (ESVAC) project, established in 2010 to collect veterinary antibiotic consumption data due owing the recent focus on livestock-associated MRSA (LA-MRSA).

Although these data related to antibiotic resistance are monitored across Europe, policies aimed at reducing MRSA infections are frequently studied at a national level [5]. However, health policy studies, such as on mandatory surveillance policies, are often discussed internationally [6]. Therefore, adopting the policies implemented by one country does not always lead to success in another. Policy evaluations are often difficult because many policies and guidelines are implemented at different times worldwide. Therefore, potentially effective national policies need to be descriptively listed and classified, using various surveillance data, to determine future implications. A comparison of the countries’ MRSA rates according to policy implementation is needed, and the differences from non-implemented countries need to be defined. Furthermore, verification of the policy of antibiotic use is needed for MRSA. LA-MRSA is an emerging topic, and therefore the use of veterinary antibiotics also needs to be investigated.

### Objective

This study aimed: (1) to list various HA- and LA-MRSA-related policies; (2) to compare the difference in MRSA incidence among countries with and without a mandatory surveillance policy; (3) to investigate the relationship between antibiotic consumption and MRSA using multiple surveillance data; and (4) to investigate the use of antibiotics for veterinary medicine.

## Methods

### Country and policy-setting

Countries were selected based on a prior study of healthcare policies among European countries [6]. We then searched for national policies and programs introduced to reduce MRSA infection, and policies were chosen based on a review article regarding MRSA policy, with opinions from healthcare professionals [7]. Further literature searches retrieved guidelines, online government reports, and publications on health policies.

### Listing of policies and distribution of MRSA rates

We listed and classified HA-MRSA-related policies implemented between 1999 and 2015. The inclusion criteria were MRSA mandatory surveillance, national guidelines, hand hygiene, antibiotic restriction, isolation rooms, and universal screening [5,8–11]. We excluded decolonization since the isolation and screening policy includes decolonization as a set, or it overlaps between the two. In addition, we searched for campaigns such as antibiotic stewardship campaigns if such policies were not implemented. We also searched for LA-MRSA-related policies similar to those for humans, such as restrictions on veterinary antibiotic and prophylactic use. We examined the policies implemented in each country aimed at reducing HA-MRSA. Furthermore, using EARS-Net annual surveillance data from 1999 to 2015, MRSA rates in each country were determined as a percentage of all *S. aureus*.

### Rates of change in MRSA incidence following implementation of mandatory surveillance

To compare MRSA rates after the implementation of mandatory surveillance policies, we assigned MRSA rates of change after adjusting for the effects of country and implementation period. We set each country at the same point, with a rate of 1.0, depending on the initial implementation year for MRSA mandatory surveillance. We also compared the difference in MRSA proportion between countries with and without MRSA mandatory surveillance policies. The rate of change was calculated by dividing the initial implementation year and the latest MRSA proportion; for countries without mandatory surveillance policies, the calculation was from the maximum rate to the latest surveillance data. The rate refers to the total numbers of laboratory screening tests and MRSA incidence.

### Antibiotic consumption and MRSA

Using ESAC-Net and EARS-Net surveillance data from 1999 to 2012, we investigated whether cephalosporin and fluoroquinolone consumption correlated with MRSA. Using the Pearson product-moment correlation coefficient, we plotted antibiotic consumption by MRSA rate from 1999 to 2012 (Spain 2000–2012 and France 2001–2012). The unit for the antibiotic consumption data was defined daily dose (DDD), a comparative unit of drug use of the roughly estimated average maintenance dose consumed per day.

### Veterinary antibiotic consumption

We used ESVAC data to investigate veterinary antibiotic consumption among seven countries between 2009 and 2015. These surveillance data were based on annual sales of veterinary antibiotics used for livestock. The sales data were normalized by livestock population according to the population correction unit (PCU), which refers to the estimated weight of livestock and slaughtered animals (1 PCU = 1 kg). PCU per country was calculated by multiplying several factors, including the number of animals slaughtered, livestock, and imports or exports for fattening or slaughter per year and country, by average weight at treatment [12].

## Results

Policies implemented for HA- and LA-MRSA varied among the seven countries included in the study (Table 1): the UK, France, Germany, Belgium, the Netherlands, Spain, and Italy.

**Table 1.** Policy implementations for healthcare-acquired methicillin-resistant *Staphylococcus aureus* (HA-MRSA) and livestock-associated methicillin-resistant *Staphylococcus aureus* (LA-MRSA) in seven countries.

	UK	France	Germany	Belgium	Netherlands	Spain	Italy
HA-MRSA; ○, *year, reference*							
Mandatory MRSA surveillance	○ 2001, 2005 [5]	○ 2001 [13]	○ 2009 [14]	○ 2006 [15]	○ (1988) [10]		
National MRSA guidelines	○ (1986) [8]	○ (1995) [16]	○ 1999 [17]	○ (1993) [18]	○ (1988) [10]	○ 2008 [19]	○ 2011[20]
Hand hygiene campaigns	○ 2004 [9]	○ 2009 [9]	○ 2008 [9]	○ 2005 [9]		○ 2006 [9]	○ 2007 [9]
Antimicrobial restrictions	* 2008 [21]	* 2002 [21]		* 2000, 2004 [21]	○ (1988) [10]	* 2006, 2007 [21]	* 2007 [21]
Single room isolation					○ (1988) [10]		
Universal screening	○ 2010, 2014 [11,22]						
LA-MRSA; ○, *year, reference*							
ESVAC mandatory reporting	○ 2005 [12]		○ 2011 [12]	○ 2006 [12]		○ 2010 [12]	○ 2011 [12]
Antimicrobial restriction	* 2012 [23]	* 2012 [24]			○ 2011 [25]		
Prophylactic use restrictions	* 2012 [24]	* 2012 [24]	○ 2010 [26]	○ 2014 [27]	○ 2011 [25]		

Data are shown as year [reference]; years in parentheses are before the study period.

*National voluntary campaign. ESVAC, European Surveillance of Veterinary Antimicrobial Consumption.

### Policies implemented in the seven European countries

Regarding HA-MRSA, an MRSA mandatory surveillance policy was implemented in the UK, France, Germany, and Belgium [5,13–15], but all countries implemented national guidelines for MRSA infection, with Italy enacting the latest in 2011 [8,10,16–20].

All countries had introduced similar hand hygiene campaigns, except for the Netherlands [9], which was the only country to implement isolation room and antibiotic restriction policies. These policies were implemented in combination with a ‘search and destroy’ policy to control MRSA infection through decolonization and strong disinfection in patient areas [10]. The UK was the first and only country to introduce a universal screening policy in 2010, mandating all patients in all healthcare facilities to be screened for MRSA when first admitted [11]. Regarding LA-MRSA, the UK, Germany, Belgium, Spain, and Italy had similar systems of mandatory reporting to ESVAC [12]. Furthermore, France, Germany, Belgium, and the Netherlands enacted similar policies or action plans restricting veterinary antibiotic or prophylactic use [24–27].

### Trend in MRSA rates following policy implementation

**Figure 1.**
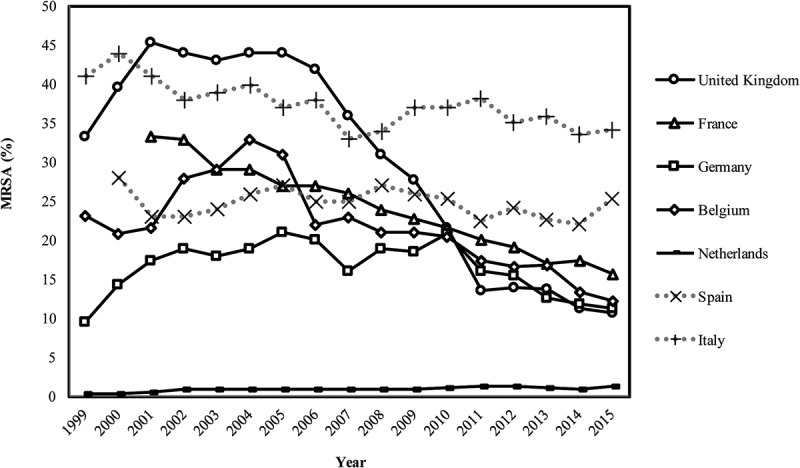
Trend in methicillin-resistant *Staphylococcus aureus* (MRSA) rates for seven countries, 1999–2015.

As shown in Figure 1, MRSA rates ranged from 1% to 45.4% across the seven countries between 1999 and 2015. Various policies were implemented to combat MRSA during different periods. Countries with more policies against MRSA (the UK, France, Belgium, Germany, and the Netherlands) showed a greater reduction in MRSA rates than those with fewer policies (Spain and Italy).

### Rates of change in MRSA following implementation of mandatory surveillance

**Figure 2.**
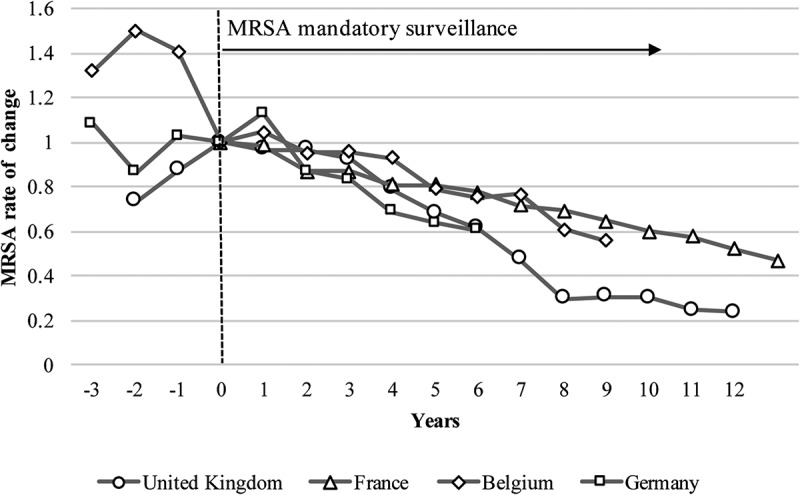
Rate of change in methicillin-resistant *Staphylococcus aureus* (MRSA) after the implementation of mandatory surveillance policies.

MRSA mandatory surveillance was implemented in 2001 in the UK and France, in 2006 in Belgium, and in 2009 in Germany [5,13–15]; the Netherlands was excluded because implementation occurred before the evaluation period [9]. We observed a decreasing rate of change following the implementation of mandatory surveillance (Figure 2).

The percentage reduction in MRSA was greater, and the 95% confidence interval narrower, for countries with mandatory surveillance policies than those without (Table 2). In addition, the rate of change was smaller for countries without such policies than those with them.

**Table 2.** Difference in proportion and rate of change among countries with and without mandatory methicillin-resistant *Staphylococcus aureus* (MRSA) surveillance policies.

	Implementation year or maximum			Latest data (2015)			Difference of proportion			Rate of change
	MRSA (*n* = 2222)	Test (*n* = 7246)	%	MRSA (*n* = 5171)	Tests (*n* = 19,046)	%	%	95% CI	*p*	%
Country with mandatory surveillance policy									< 0.001	
UK	676	1488	45.4	298	2757	10.8	34.6	(29.8 to –38.8)		76% ↓
France	572	1714	33.4	869	5535	15.7	17.7	(15.3 to 20.2)		53% ↓
Belgium	189	858	22.0	112	913	12.3	9.7	(6.1 to 13.3)		44% ↓
Germany	350	1894	18.5	546	4871	11.2	7.3	(5.3 to 9.3)		39% ↓
Country without mandatory surveillance policy										
Spain	234	836	28.0	498	1970	25.3	2.7	(−0.9 to 6.4)	0.13	10% ↓
Italy	201	456	44.0	1023	3000	34.1	9.9	(5.0 to 14.9)	< 0.001	23% ↓

The chi-squared test was applied for comparing two proportions of implementation year or at the maximum to the latest data (2015).

### Antibiotic consumption and MRSA

**Figure 3.**
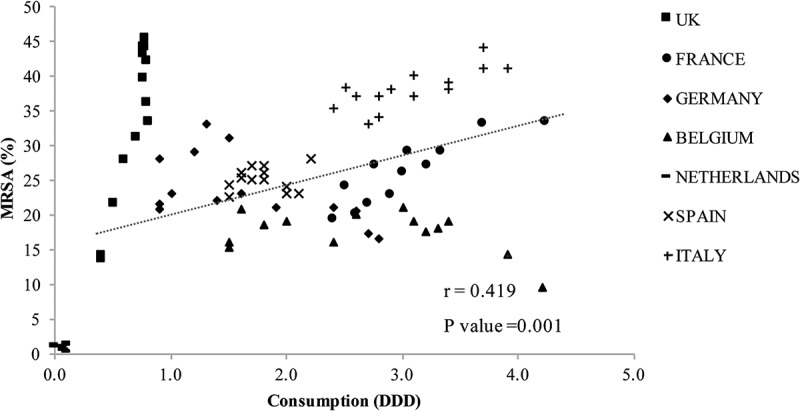
Cephalosporin consumption and methicillin-resistant *Staphylococcus aureus* (MRSA). DDD, defined daily dose.

**Figure 4.**
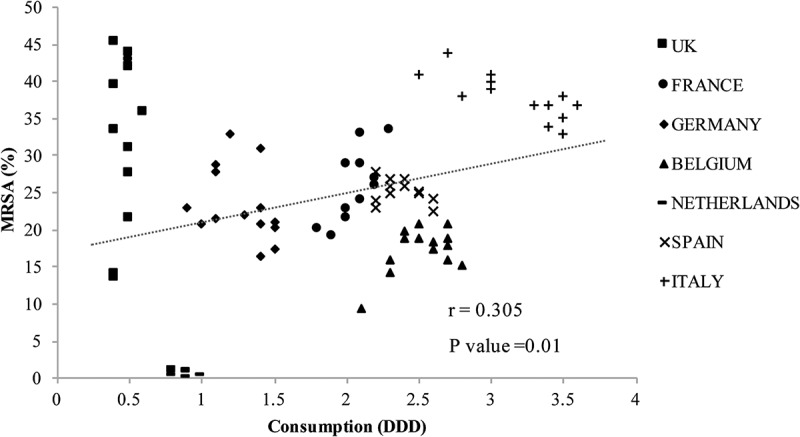
Fluoroquinolone consumption and methicillin-resistant *Staphylococcus aureus* (MRSA). DDD, defined daily dose.

The analysis results for antibiotic consumption and MRSA rates revealed a moderate correlation for cephalosporin (Figure 3) and a weak correlation for fluoroquinolone (Figure 4). The UK, Germany, and the Netherlands had a lower consumption of cephalosporin and fluoroquinolone compared with other countries, but the UK and Germany had relatively high MRSA rates. Italy had the highest cephalosporin and fluoroquinolone consumption and high MRSA rates.

### Annual sales of antibiotics for veterinary medicine

**Figure 5.**
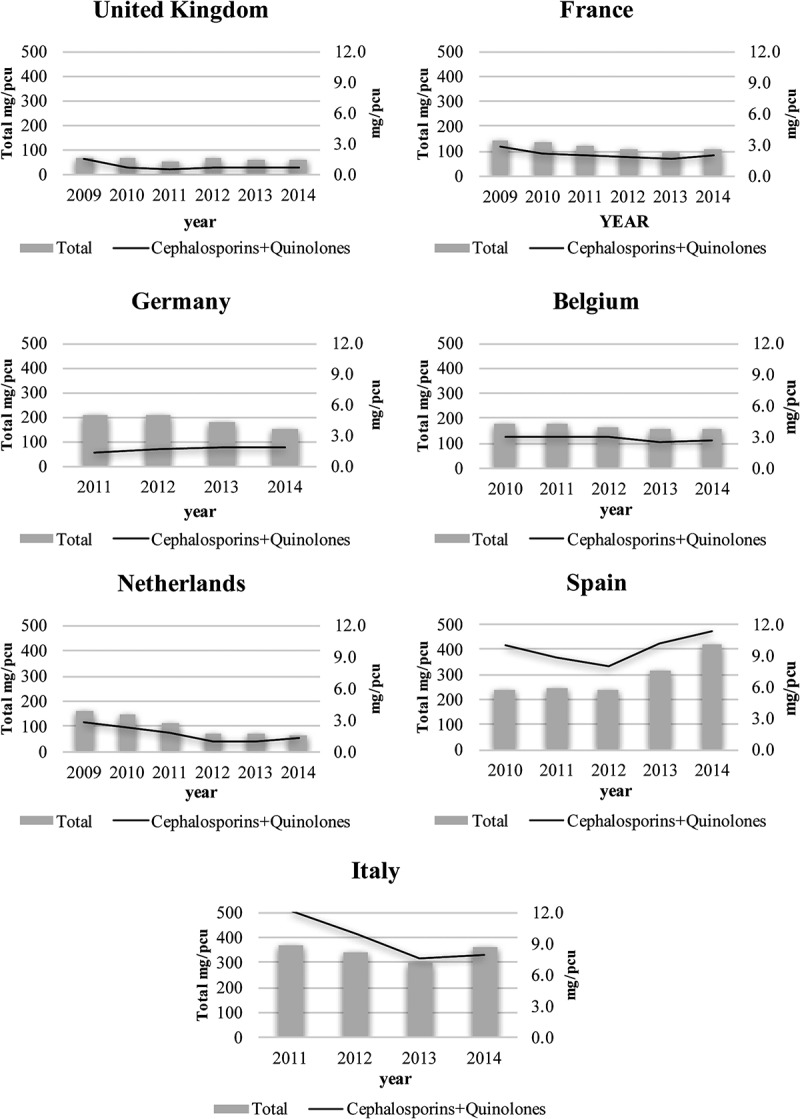
Trend in livestock antibiotic consumption for seven countries.

Between 2009 and 2014, the mean annual sales of veterinary cephalosporin and quinolone were lowest in the UK (0.8 mg/PCU) and highest in Spain (9.7 mg/PCU) (Table 3). The UK also had the lowest quinolone sales, while the Netherlands had the lowest cephalosporin sales. Spain was the only country to increase not only cephalosporin and quinolone but also total antibiotic sales (Figure 5).

**Table 3.** Consumption of veterinary antimicrobial agents in seven countries, 2009–2014.

	UK	France	Germany	Belgium	Netherlands	Spain	Italy	All countries
Sales of veterinary antimicrobials (mg/PCU)								
Cephalosporins	0.5 (0.1–0.8)	0.6 (0.4–0.9)	0.5 (0.4–0.5)	0.6 (0.5–0.6)	0.2 (0.1–0.3)	0.5 (0.2–0.7)	0.7 (0.6–0.8)	0.5 (0.4–0.6)
Quinolones	0.3 (0.3–0.4)	1.5 (1.3–1.7)	1.2 (0.9–1.6)	2.3 (2.0–2.5)	1.5 (0.9–2.1)	9.2 (7.9–10.6)	8.7 (5.8–11.6)	3.3 (1.0–1.7)
Cephalosporins + quinolones	0.8 (0.4–1.1)	2.1 (1.7–2.5)	1.7 (1.3–2.0)	2.9 (2.6–3.1)	1.7 (1.0–2.4)	9.7 (8.6–11.1)	9.4 (6.4–12.3)	3.7 (2.6–4.9)
All classes	62.8 (60.9–64.8)	117.2 (111.5–122.8)	186.0 (177.7–232.8)	166.6 (163.5–169.7)	106.3 (93.3–119.3)	293.6 (270.3–316.9)	343.3 (334.5–352.1)	170.4 (137.2–203.7)

Data are shown as mean (95% confidence interval).

Data from Belgium, Germany, Spain and Italy are from 2010 or 2011.

## Discussion

We have listed and classified the LA-MRSA and HA-MRSA-related policies implemented by seven European countries using three different sets of surveillance data. Although we could not define the causality of each policy, contextual descriptions allow us to identify each country’s situation, with classification as the first step for cross-national variations [28,29]. To resolve such causality issues between cross-national comparison and health policies, multilevel modeling using a Bayesian approach may be preferred [30]. Nevertheless, since each country’s background influences policy implementation, determining other countries’ implementation status is important; we therefore discuss each potential policy that might have affected MRSA.

In this study, the downward trend in MRSA incidence was more substantial in countries with mandatory surveillance policies than in those without. Although we could not define the difference between the countries with and without mandatory policies, this study may be the first step towards better control of MRSA for the latter countries. Even though MRSA rates gradually decreased in all the countries, there may be different effects depending on the strictness and frequency with which policies are implemented. For instance, the UK implemented MRSA mandatory surveillance in 2001 and enhanced it in 2005 [5], enabling the collection of comprehensive data on patient backgrounds and history, and possibly reducing MRSA bacteremia in the following year [5]. In Belgium, several voluntary reporting campaigns were implemented between 1994 and 2003 [14], which may have already started to affect the MRSA rates. These campaigns, other related policies, and simply the nature of trend may have had similar effects to the other countries. Therefore, further study is needed to define causation, but such policies can act as templates for countries that are yet to implement surveillance.

Regarding other policies, all countries had individual national guidelines aimed at reducing MRSA infections. Every country had similar guidelines, but differed according to whether the country had implemented an MRSA mandatory surveillance policy, single room isolation, or universal screening. Although varying levels of guidance and physician compliance make it difficult to determine a policy’s effectiveness, national guidelines provide the most reliable evidence currently available for effective treatment and are therefore necessary for reducing MRSA infections [31,32].

Many countries had initiated hand hygiene campaigns following the World Health Organization’s global ‘Clean Care is Safer Care’ program in 2005 [9]. Alcohol-based hand rubs in healthcare facilities do reduce MRSA infections, but the effectiveness of campaigns depends on the compliance of healthcare workers [9,31]. Therefore, the impact of these policies on MRSA infections requires further investigation.

This study found that an isolation room policy was implemented only in the Netherlands. Van Rijen et al. [33] reported that isolation rooms and screening were cost-effective in countries with low MRSA infection rates, and as shown in Figure 1, the Dutch ‘search and destroy’ policy seems to have controlled MRSA rates. Although an isolation room policy was not implemented throughout the UK, Scotland has had such a policy for 10 years [34,35]. In Germany, the Commission for Hospital Hygiene and Infection Prevention recommended isolation and screening for MRSA [36], but not as strictly as the ‘search and destroy’ policy of the Netherlands [10].

Nationwide antibiotic restriction policies or campaigns were effective in the UK, France, Belgium, the Netherlands, and Italy [21,37,38], except that despite reduced antibiotic consumption in Italy, awareness of antibiotic resistance had not increased [37]. Spain has conducted a campaign since 2006 and reduced antibiotic consumption, but many antibiotics are still sold without a prescription in some areas [39,40].

The UK was the only country to implement a universal screening policy; however, Robotham et al. [41] found that the policy was not cost-effective, and the UK government changed the policy to screening mainly high-risk patients from 2014 [22]. Although previous universal screening was not cost-effective, its long-term implications should nevertheless be assessed.

Excessive antibiotic use is believed to increase antimicrobial resistance, and cephalosporin and fluoroquinolone consumption correlate with MRSA incidence [3,4], as verified in this study. This finding could differ in other countries; therefore, an analysis of more countries is needed.

Antibiotic use is also considered a cause of LA-MRSA in veterinary medicine [42]. Although human-to-human transmission of LA-MRSA infection is much rarer than HA-MRSA, it may also be acquired by handling contaminated meat products such as poultry, allowing the possibility of spreading LA-MRSA [43]. In this study, veterinary antibiotic consumption was not compared with LA-MRSA incidence, but since LA-MRSA may be acquired by humans from its use, we investigated the consumption between countries. Moves towards restricting antibiotic use in treating livestock are being considered. Following the sixteenth amendment to the Medicine Act in 2010, Germany implemented stricter regulations on antibiotic use, such as restricting prophylactic use and advising only therapeutic use [24]. The Health Council of the Netherlands also enacted a policy to limit antibiotic use, which had the most effective results (Figure 5) [26].

Our study has several limitations that warrant attention. The study relied on raw national surveillance data, which may not be accurate. EARS-Net and ESAC-Net data do not cover all healthcare facilities and countries by population density, and so each country’s MRSA rate may not be accurately distributed: the results could be overestimated or underestimated for countries with a low participation rate. However, national surveillance data have the highest degree of validation available for international-level research. Furthermore, the population coverage of countries chosen for this study was over 90% for all ESAC-Net antibiotic consumption data [44].

The policies chosen for our study may not be the most suitable ones. Although they may have influenced the decline in MRSA rates, other policies and campaigns related to prevention, decolonization, and rational antibiotic stewardship may have also had an effect, as well as contact precautions and prophylactic glycopeptide antibiotics. Other potential factors may affect the MRSA rates, such as the development of medical devices for screening tests and improvement of treatment. In this study, policy implementation in the UK focused only on England, but the UK comprises four countries, each with different legislation, financial penalties, and reduction targets for MRSA, meaning that results may vary by country.

Furthermore, each country has a different economic situation with restrictive budgets for MRSA prevention, along with different virulence and epidemiology of MRSA strains. These differences, which may have affected the trend, were not considered in this study. It is also possible that countries such as France and the Netherlands had a downward trend in MRSA before the study period, but regional policies or campaigns from that time were not examined. The cost-effectiveness of individual policies should also be considered, but these data were unavailable and could not be investigated. Although we could not resolve the potential limitation of omitted policies, policy selection using prior research was probably sufficient for the initial investigation.

We listed and classified LA- and HA-MRSA-related policies in seven European countries along with multiple surveillance data. Although causation could not be defined, mandatory surveillance may be an effective policy to reduce MRSA rates. Further investigations are needed to evaluate each policy.
